# Phylogeographic Aspects of Bat Lyssaviruses in Europe: A Review

**DOI:** 10.3390/pathogens12091089

**Published:** 2023-08-27

**Authors:** Heliana Dundarova, Nadya Ivanova-Aleksandrova, Sarka Bednarikova, Irina Georgieva, Krasimir Kirov, Kalina Miteva, Boyko Neov, Peter Ostoich, Jiri Pikula, Jan Zukal, Peter Hristov

**Affiliations:** 1 Institute of Biodiversity and Ecosystem Research, Bulgarian Academy of Sciences, 1 Tsar Osvoboditel Blvd., 1000 Sofia, Bulgaria; 2 National Centre of Infectious and Parasitic Diseases, 26 Yanko Sakazov Blvd., 1504 Sofia, Bulgaria; 3 Department of Ecology and Diseases of Zoo Animals, Game, Fish and Bees, University of Veterinary Sciences Brno, Palackého tř. 1946/1, 612 42 Brno, Czech Republic; 4 Faculty of Biology, University of Plovdiv “Paisii Hilendarski”, 24 Tzar Assen Str., 4000 Plovdiv, Bulgaria; 5 Institute of Vertebrate Biology, Czech Academy of Sciences, Květná 8, 603 65 Brno, Czech Republic

**Keywords:** lyssavirus phylogroups, Chiroptera, evolution, transition routes, health

## Abstract

During the last few decades, bat lyssaviruses have become the topic of intensive molecular and epidemiological investigations. Since ancient times, rhabdoviruses have caused fatal encephalitis in humans which has led to research into effective strategies for their eradication. Modelling of potential future cross-species virus transmissions forms a substantial component of the recent infection biology of rabies. In this article, we summarise the available data on the phylogeography of both bats and lyssaviruses in Europe and the adjacent reg ions, especially in the contact zone between the Palearctic and Ethiopian realms. Within these zones, three bat families are present with high potential for cross-species transmission and the spread of lyssaviruses in Phylogroup II to Europe (part of the western Palearctic). The lack of effective therapies for rabies viruses in Phylogroup II and the most divergent lyssaviruses generates impetus for additional phylogenetic and virological research within this geographical region.

## 1. Introduction

The Order Chiroptera has a Laurasiatherian origin (“laurasian beasts”), evolved between 50 and 70 million years ago (MYA), and has undergone rapid diversification [1,2]. Due to their capabilities of self-powered flight and echolocation, bats [3] comprise over 20%, or more than 1460 species, of all modern mammals and are globally distributed, with the exception of the extreme polar regions [4]. They have many characteristics that differentiate them from other mammalian species, such as their unique physiology [5,6], metabolism [7], and immune system [2,8,9]. These features make them a suitable reservoir for viral zoonoses [4,10,11] and more than 200 viruses have been isolated from or detected in bats [12,13,14]. The order comprises 45 species in Europe [15] from two superfamilies, the Rhinolophoidea and Vespertilionoidea [16], representing a natural reservoir of RNA-viruses (Table 1). 

Viruses from 11 families have been isolated on the continent [17] and bat lyssaviruses in Europe (family *Rhabdoviridae*) have been the subject of detailed reviews [18,19,20,21]. Lyssaviruses are a genus of negative-sense single-strand RNA viruses in the family *Rhabdoviridae*, subfamily *Alpharhabdovirinae*. Notably, they are members of the order *Mononegavirales*, which includes other prominent zoonotic pathogens such as filoviruses (Ebola, Marburg, etc.) and the neurotropic *Bornaviridae* [22]. Based on genetic divergence, lyssaviruses are classified into 21 different viral species. Recently, several putative new lyssaviruses were published [23,24,25,26]. Apart from the Mokola virus (MOKV) and Ikoma lyssavirus (IKOV), which have rodents and African civets as a reservoir, respectively [25,27,28], the rest of the lyssaviruses can be transmitted by Chiroptera [27,29]. According to the most recent ICTV report [24], lyssavirus names are provided here followed by the traditional abbreviations used to identify their isolates: rabies virus (RABV), Aravan virus (ARAV), Australian bat lyssavirus (ABLV), Bokeloh bat lyssavirus (BBLV), Duvenhage virus (DUVV), European bat lyssavirus 1 (EBLV-1), European bat lyssavirus 2 (EBLV-2), Gannoruwa bat lyssavirus (GBLV), Ikoma lyssavirus (IKOV), Irkut virus (IRKV), Khujand virus (KHUV), Lagos bat virus (LBV), Lleida bat lyssavirus (LLEBV), Mokola virus (MOKV), Shimoni bat virus (SHIBV), Kotalahti bat lyssavirus (KBLV), Divača bat lyssavirus (DBLV)**,** West Caucasian bat virus (WCBV), Matlo bat lyssavirus (MBLV), and Lyssavirus *Formosa*, which includes Taiwan bat lyssavirus 1 (TWBLV-1) and Taiwan bat lyssavirus 2 (TWBLV-2) [21,24,30,31,32,33,34,35]. In fact, KBLV and MBLV are only tentative lyssaviruses. The current study aims to review the evolution, phylogeography, and transmission routes of bat lyssaviruses in Europe.

**Table 1:** European bat species with identified lyssaviruses and their IUCN conservation status. Abbreviations: EN: Endangered—very high risk of extinction in the wild; VU: Vulnerable—high risk of extinction in the wild; NT: Near Threatened—likely to become threatened in the near future; LC: Least Concern—does not qualify for a more at-risk category. Widespread and abundant taxa are included in this category; Data Deficient—inadequate information to make a direct, or indirect, assessment of its risk of extinction based on its distribution and/or population status; N/A: not assessed.

Bat Species	IUCN Status	Bat Identification	Virus Detection	European Bat Lyssaviruses														References
				EBLV-1		EBLV-2		BBLV		KBLV		DBLV		LLEBV		WCBV		
				Spill over Infection	Host Species	Spill over Infection	Host Species	Spill over Infection	Host Species	Spill over Infection	Host Species	Spill over Infection	Host Species	Spill over Infection	Host Species	Spill over Infection	Host Species	
family Rhinolophidae																		
*Rhinolophus ferrumequinum*	NT	morphology	FAT, RT-PCR, RFFIT, mFAVNt		+													[36,37]
family Vespertilionidae																		
*Barbastella barbastellus*	VU	morphology	mFAVNt		+													[37]
*Eptesicus isabellinus*	N/A	morphology	RT-PCR		+													[38,39]
*Eptesicus serotinus*	LC	*cyt b*	FAT, RT-PCR, RFFIT	+	+													[36,38,39,40,41,42,43,44,45,46,47,48,49]
*Myotis blythii*	NT	morphology	mFAVNt		+													[37]
*Myotis brandtii*	LC	*nd1*, *cyt b*	FAT, RT-PCR								+							[24,45]
*Myotis capaccinii*	VU	morphology	FAT, RFFIT, RTCIT, RT-PCR										+					[35]
*Myotis dasycneme*	NT	morphology	RT-PCR				+											[41,45,49]
*Myotis daubentonii*	LC	*cyt b*	RT-PCR			+	+											[41,45,46,47,48,49]
*Myotis myotis*	LC	*cyt b*	FAT, RT-PCR, RFFIT		+													[36,45,48]
*Myotis nattereri*	LC	*cyt b*	FAT, RT-PCR, RFFIT		+				+									[18,45,50,51,52]
*Nyctalus noctula*	LC	*cyt b*	FAT, RT-PCR		+													[45,48]
*Pipistrellus nathusii*	LC	*cyt b*	FAT, RT-qPCR, RT-PCR, RTCIT		+													[45,48]
*Pipistrellus pipistrellus*	LC	*cyt b*	FAT, RT-qPCR, RT-PCR, RTCIT		+													[45,48]
*Plecotus auritus*	LC	*cyt b*	FAT, RT-qPCR, RT-PCR, RTCIT		+													[45,48]
*Vespertilio murinus*	LC	*cyt b*	RT-PCR		+													[41,45]
family Miniopteridae																		
*Miniopterus schreibersii*	NT	morphology	FAT, RT-PCR, RFFIT, mFAVNt		+										+	+	+	[36,37,53]
family Molossidae																		
*Tadarida teniotis*	LC	morphology	FAT+		+													[36]
family Pteropodidae																		
*Rousettus aegyptiacus*	N/A (EN?)	morphology	FAT, RT-PCR, RFFIT		+													[54]

## 2. Origin, Evolution, and Geographic Distribution of Bat Lyssaviruses

Despite the greater diversity of African lyssaviruses [55], Hayman et al. [56] assumed that they have a Palearctic origin and challenged “Out of Africa” hypothesis. The Lyssaviruses’ most recent common ancestor (MRCA) evolved from an insect rhabdovirus between 7000 and 11,000 years ago [30,57,58] which was transmitted to representatives of the order Chiroptera and spread globally [57,59]. According to Rupprecht et al. [30], Africa is the most likely home to the ancestors of taxa within the Genus *Lyssavirus*, family *Rhabdoviridae*. According to this review, a large number of different lyssaviruses co-evolved with bats as ultimate reservoirs over millions of years. On the other hand, Velasco-Villa et al. [60] argue that in the Western Hemisphere before the arrival of the first European colonizers, rabies virus was present only in bats and so-called mesocarnivores (canids, raccoons, skunks, etc.). It is assumed that all mammals are susceptible to infection with the rabies virus. However, it is most possible that lyssaviruses will never be eradicated due to their presence in chiropteran hosts. 

Lyssaviruses have undergone purifying selection followed by a neutral evolution of the viral genomes [61]. The low rate of nonsynonymous evolution of lyssaviruses is probably the result of constraints imposed by the need to replicate in multiple cell types (muscle, peripheral and central nervous systems, and salivary glands) within the host, which in turn boosts cross-species transmission (e.g., different groups of mammals), or because viral proteins are not subject to immune selection, which means existing lyssaviruses are well adapted to their reservoir [62,63]. 

The host switching of the classic rabies lyssavirus (RABV) from bats to other mammals is estimated to have occurred 800 to 1400 years ago, which does not explain the timing of the oldest putative human rabies cases, estimated to have circulated 4000 years ago in ancient Mesopotamia [64,65]. A possible explanation is that the Mesopotamian RABV lineage disappeared as a consequence of genetic drift (loss of polymorphism) or its high fatality rates [64]. According to Rupprecht et al. [66] and Badrane et al. [67], bats are the primary evolutionary host of rabies viruses as a reservoir of all existing lyssaviruses except MOKV and IKOV, whereas other mammals and humans only maintain several lineages of RABV, including the extinct Mesopotamian strain [30,64,68].

**Figure 1:**
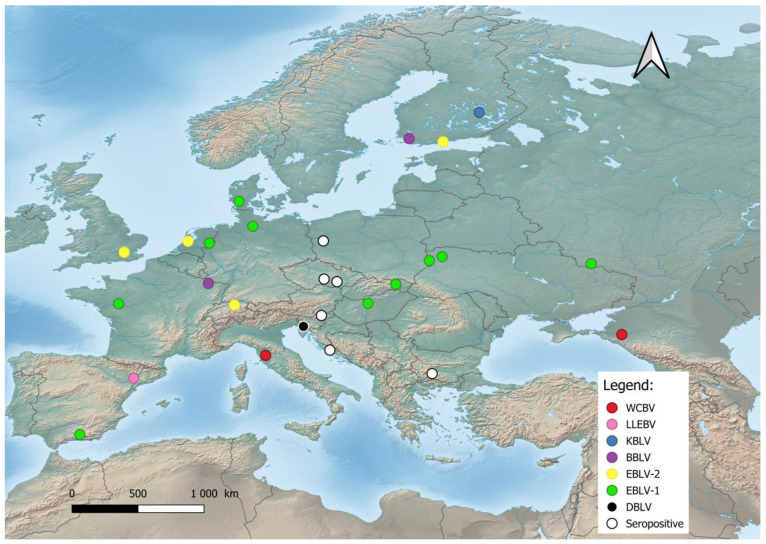
Distribution of bat lyssaviruses in Europe. Abbreviations used: WCBV—West Caucasian bat lyssavirus; LLEBV—Lleida bat lyssavirus; KBLV—Kotalahti bat lyssavirus; BBLV—Bokeloh bat lyssavirus; EBLV-1—European bat lyssavirus 1; EBLV-2—European bat lyssavirus 2; DBLV—Divača bat lyssavirus, Seropositive—Seropositive Blood samples.

In Europe, bat lyssaviruses (Figure 1) were detected in the United Kingdom, the Netherlands, Finland, Denmark, Poland, Czech Republic, Germany, Switzerland, France, Spain, Hungary, Italy, Slovenia, Croatia, Bulgaria, Ukraine, and Russia [19,21,35,38,69,70,71]. During the last two decades, previously unknown lyssaviruses were isolated as follows: WCBV in 2002 on the European side of the Caucasus Mts. [72], BBLV in 2010 from Germany [50], LLEBV in 2011 from Spain [73], KBLV in 2017 from Finland [23], and DBLV in 2014 from Slovenia [35]. 

The most frequent lineages are EBLV-1, first reported in 1955 from Germany, and EBLV-2, isolated in 1985 in Switzerland [38,68]. EBLV-1 is exclusively detected in Serotine bats (*Eptesicus serotinus*), while EBLV-2 is mainly found in Daubenton’s bats (*Myotis daubentonii*). EBLV-1 is present in two forms: EBLV-1a and EBLV-1b. EBLV-1a displays a wide geographical distribution between France and Russia with phylogenetic homogeneity—an indication of extensive dispersal by bats [20,41]. Resent research has shown that EBLV-1 is associated with the bat *E*. *serotinus* of the mountainous parts of Southern Europe, such as the French Alps or the Iberian Peninsula [39]. EBLV-1 demonstrates the risk of spillover because of its host’s close phylogenetic relation with a different bat, the *E*. *isabellinus*. The phylogenetic analysis of nine EBLV-1 strains of *E*. *serotinus* distributed in the south of the Pyrenees revealed that two of them are closely related to EBLV-1a sequences from Southern France, i.e., this group expanded to Northern Spain. The results of the conducted research give the authors reason to assume the expansion of the EBLV-1a subtype across southern France, with a very recent arrival to the Iberian Peninsula, i.e., a current southwards dissemination [38]. In contrast, EBLV-1b is distributed between Spain and Poland with a well-defined geographic structure, indicating restricted contact between bat populations [20,38]. Therefore EBLV-1b had the potential to spread southwards according to the *E. isabellinus* distribution. The lineage of EBLV-1 is presumed to have arisen 500 to 750 years ago and has a relatively recent origin [41]. Conversely, the lineage of EBLV-2 is dated to more than 8000 years ago, with current establishment in Europe within the last 2000 years. [74]. EBLV-2 has been reported in Western Europe and is also represented by two forms: EBLV-2a and EBLV-2b [69,75]. The first occurs in the United Kingdom, Netherlands, Germany, Switzerland, and Denmark, while the second includes the Finnish EBLV-2 strains and a strain from Switzerland [74], where the divergence of the Finnish strains from the Swiss strain occurred within the last 200 years [74].

## 3. Virion Structure and Genome

Rhabdoviruses (family *Rhabdoviridae*) have a characteristic bullet-shaped virion morphology, with an envelope derived from the plasma membrane of the infected host cell and approximate dimensions of 60–110 nm × 130–250 nm, which distinguishes them from other taxa in the order *Mononegavirales: Bornaviridae*, *Filoviridae*, and *Paramyxoviridae*. They include a 11.9–12.3 kb long non-segmented, linear, single-strand RNA genome. The basic genome includes five genes that encode (from 3′ to 5′) the nucleoprotein (or nucleocapsid protein, N), phosphoproteins (P), matrix protein (M), glycoprotein (G), and large protein (L, RNA—dependent RNA polymerase) [76,77]. 

Open reading frames known as ORFs present an ancestral pseudogene [78] which is used for studying virus–host interactions in WCBV [59] due to the outstanding size, which is 40% larger than in other bat lyssaviruses [79]. It has been found that in some rhabdoviruses very long non-coding regions (up to 749 nt) were present either within or between transcriptional units [59]. This region seems to serve as a resource for the de novo emergence of genes which may be related to elucidating the taxonomy, phylogeny, and evolution of lyssaviruses. This is most likely to occur when ORFs are present in transcribed non-coding regions (UTRs) such as in the so-called ‘pseudogene ψ region’ of WCBV, which is unique to an ORF of 180 nt. The de novo creation of genes in non-transcribed intergenic regions (IGRs), as well as those present in the G-L gene junctions of various hapavirus, is associated with prior or simultaneous evolution of new or modified transcriptional control sequences. In the trend towards increasing genome size and complexity in rhabdoviruses, the loss of a gene and/or genes is also likely to have occurred periodically, which may also be evolutionarily determined in the family Rhabdoviridae [59].

## 4. Phylogeny of Bat Lyssaviruses

**Figure 2:**
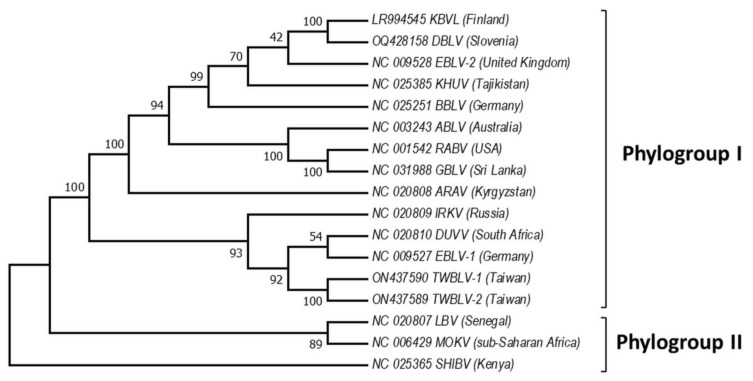
Phylogeny of bat lyssaviruses. The N + P + M + G + L coding regions of representative reference sequences of lyssaviruses used in the analysis were derived from Genbank. The evolutionary history was inferred by using the Maximum Likelihood method and General Time Reversible model [91]. There were a total of 568 positions in the final dataset. Evolutionary analyses were conducted in MEGA X [92]. Virus names are: RABV—rabies virus, ARAV—Aravan virus, ABLV—Australian bat lyssavirus, BBLV—Bokeloh bat lyssavirus, DUVV—Duvenhage virus, EBLV-1—European bat lyssavirus 1, EBLV-2—European bat lyssavirus 2, GBLV—Gannoruwa bat lyssavirus, IKOV—Ikoma virus, IRKV—Irkut virus, KHUV—Khujand virus, LBV—Lagos bat virus, MOKV—Mokola virus, SHIBV—Shimoni bat virus, KBVL—Kotalahti bat lyssavirus, DBLV—Divača bat lyssavirus, TWBLV-1—Taiwan bat lyssavirus 1, and TWBLV-2—Taiwan bat lyssavirus 2.

Based on the sequence analysis of the lyssavirus N gene, serologic cross-reactivity and pathogenicity bat lyssaviruses are divided into two phylogroups [67,80,81,82], https://ictv.global/report/chapter/rhabdoviridae/rhabdoviridae/lyssavirus and an unresolved but widely adopted third phylogroup [83,84], https://www.who-rabies-bulletin.org/site-page/classification which might contain some of the most divergent lyssaviruses (Figure 2). For simplicity, we used 17 reference sequences of N + P + M + G + L [32,33,35,50,72,80,82,85,86,87,88,89,90] genes available in GenBank for our phylogenetic analysis (Supplementary Table S1). European viruses are included in Phylogroups I and group of lyssaviruses, which are highly divergent. Phylogroup II is discussed only as a potential scenario for cross-species bat transmission. 

Phylogroup I includes all these lyssaviruses RABV, ARAV, ABLV, BBLV, DUVV, EBLV-1, EBLV-2, GBLV, IRKV, KBLV, DBLV, KHUV, TWBLV-1, and TWBLV-2, whereas LBV, MOKV, and SHIBV form Phylogroup II [23,31,34,35,44,45,85,86]. Phylogenetically, the most divergent lyssaviruses LLEBV, IKOV, WCBV, and MBLV appear related [27,50,73,93]. Phylogroup I is divided into two major groups: the first includes the Palearctic lyssaviruses IRKV, EBLV-1, TWBLV-1, TWBLV-2 and African DUVV lyssaviruses and the second ARAV, BBLV, KHUV, and EBLV-2 which are also lyssaviruses with Palearctic distribution, as well as Australian—ABLV, Oriental—GBLV, and American—RABV [57]. Interestingly, EBLV-1 is most closely related to DUVV and IRKV, while EBLV-2 to KBLV, KHUV, and BBLV [30,32]. Based on the close phylogenetic relation between EBLV-1 and DUVV lyssaviruses [49], it is hypothesized that EBLV-1 originated in North Africa and spread to Europe (Iberian Peninsula) via the Strait of Gibraltar. However, Hayman et al. [13] present phylogenetic evidence based on the rabies N gene sequences that EBLV-1 and DUVV share a common ancestor with IRKV (isolate from Russia) and both have been transferred to Africa from the Palearctic region, and Europe in particular. Phylogenetic relationships in the most divergent lyssaviruses demonstrate close phylogenetic relatedness between the LLEBV virus from Spain, sub-Saharan Africa MBLV with the Eurasian WCBV and the African IKOV lyssavirus [34,56,94]. Genetically, LLEBV is more closely related to IKOV than to WCBV, in contrast with MBLV [34].

For a better understanding of lyssavirus phylogeny and their current distributions, a closer look at their bat species reservoirs is required. Generally, morphological keys such as Dietz et al. [95] are widely used for bat identification. On the other hand, morphological identification from carcasses can be limited due to the state of decomposition or nearly indistinguishable morphological features in juvenile bats and can lead to misidentifications [96]. Therefore, genetic markers are highly required due to their role for precise bat taxonomic clarification especially in cryptic species complexes, e.g., Çoraman et al. [97] and De Benedictis et al. [98]. Genomic and mitochondrial analyses have placed bats into two suborders: Yinpterochiroptera—including the five families in the superfamily Rhinolophoidea plus the flying foxes—Pteropodidae, and Yangochiroptera—including the three superfamilies: Emballonuroidea, Vespertilionoidea, and Noctilionoidae, comprising a total of 13 families. Two superfamilies (Rhinolophoidea and Vespertilionoidea) are of particular interest in Europe because their representatives are the main reservoir of lyssaviruses. The greater horseshoe bat (*Rhinolophus ferrumequinum*) (Rhinolophidae, Rhinolophoidea) and the Vespertilionoidea species Greater mouse-eared bat (*Myotis myotis)*, Lesser mouse-eared bat (*M. blythii*), Natterer’s bat (*M. nattereri*), Serotine bat (*Eptesicus serotinus*), Meridional serotine (*E. isabellinus*), Common pipistrelle (*Pipistrellus pipistrellus*), Nathusius’s pipistrelle (*P. nathusii*), Brown long-eared bat (*Plecotus auritus*), Common noctule (*Nyctalus noctula*), Parti-coloured bat (*Vesperilio murinus*) (Vespertilionidae), Common bent-wing bat (*Miniopterus schreibersii*) (Miniopteridae), and European free-tailed bat (*Tadarida teniotis*) (Molossidae) have all been documented as being infected by EBLV-1 [36,37,38,40,71]. The virus was also isolated from the Egyptian fruit bat (*Rousettus aegyptiacus*) (Pteropodidae) in a Dutch zoo [53]. Regardless of the high number of bat hosts recorded for EBLV-1, EBLV-2 is restricted to *Myotis daubentonii* and *M. dasycneme* [38,68,69]. KBLV was found only in *Myotis brandtii* [23], BBLV only in *M. nattereri* [52,99], and DBLV only in *M. capacinii* [35]. For comparison, from those bat species, virus serological detection is provided on 15 bats (*R. ferrumequinum*, *B. barbastellus*, *E. serotinus*, *M. blythii*, *M. brandtii*, *M. capaccinii*, *M. myotis*, *M. nattereri*, *N. noctule*, *P. nathusii*, *P. pipistrellus*, *P. auratus*, *M. schreibersii*, *T. teniotis*, *R. aegyptiacus*), identification of viral species affiliation on 16 bats (*R. ferrumequinum*, *E. isabellinus*, *E. serotinus*, *M. brandtii*, *M. capaccinii M. dasycneme*, *M. daubentoniid*, *M. myotis*, *M. nattereri*, *N. noctule*, *P. nathusii*, *P. pipistrellus*, *P. auratus*, *V. murinus*, *M. schreibersii*, *R. aegyptiacus*) and both identified in 12 bat species (*R. ferrumequinum*, *E. serotinus*, *M. brandtii*, *M. capaccinii*, *M. myotis*, *M. nattereri*, *N. noctule*, *P. nathusii*, *P. pipistrellus*, *P. auritus*, *M. schreibersii*, *R. aegyptiacus*), see Table 1. 

However, the phylogeny of the Natterer’s bat group is more complex. In Europe, *M. nattereri* is composed of *M. escalerai* (Iberia), *M*. species A (Italy and parts of the Pyrenees), *M*. species B (Northwest Africa), *M*. species C (Corsica), and the nominal form *M. nattereri* present across the rest of Europe [52,100,101]. According to Eggerbauer et al. [102], BBLV-positive bats in Germany and France were of the nominal form. Çoraman et al. [103] provided a detailed phylogenetic analysis and reported signs of repeated hybridization between the Natterer’s bat lineages [103], with southern France a probable contact zone between different *M*. species and *M. nattereri*. Additional research could reveal the potential of BBLV to spread to other Natterer’s bat lineages.

The Common bent-wing bat (*Miniopterus schreibersii*) has been proven to be a host to IRKV and DUVV from Phylogroup I and most divergent WCBV and LLEBV. The species seems to be a universal reservoir for both phylogroups not only in Europe but also in Asia and Africa [56,104], due to the fact that *M. schreibersii* is a strictly cave-dwelling species [95] capable of long-distance migration [105]. The most abundant European lyssavirus group, EBLV-1, has still not been detected in the Common bent-wing bat despite its close phylogenetic relationship to DUVV.

## 5. Transmission Routes of Bat Lyssaviruses

### 5.1. Bat Intra- and Cross-Species Transmission

The main transmission route of rabies viruses is via a bite from the host and the virions released into the saliva during the clinical period of rabies and/or during the end of the incubation period [106,107,108]. All lineages from Phylogroup I are transmitted by bats whereas the classical rabies virus (RABV) has evolved to spread via carnivores and through bat species restricted to the Americas [21]. In Europe, EBLV-1 is the most prevalent lyssavirus among the Chiroptera due to bat colony sizes, species richness, and the presence of migratory species. Colombi et al. [109], suggested that in the roost, the main factor for virus spillover is the large number of species and their individual mobility. However, roost sizes of less than approximately 200 individuals are not enough for efficient maintenance of the lyssavirus infection [110]. On the other hand, migrant species such as *Pipistrellus nathusii*, *Nyctalus noctula*, *N. leisleri,* and *Miniopterus schreibersi* can play a key role for the dispersal of EBLV-1 in Europe, and *M. schreibersi* also for WCBV and LLEBV [84,105,111,112]. These species are capable of flying long distances between summer and winter roosts and increasing the lyssavirus geographical range via cross-species transition is an expected scenario. Consequently, seasonal bat movements between hibernacula, breeding, and mating sites are an important prerequisite for successful virus cross-species spillover. For instance, hibernation is characterized by decreased activity in the bat immune system and metabolism [113,114], thereby extending incubation periods and allowing virus persistence between transmission periods. Based on the transcriptomic responses of bat cells to EBLV-1 Constantine [115], it is concluded that the lack of bat cell reaction to infection in conditions simulating hibernation may contribute to the virus tolerance or persistence in bats. In addition, long hibernation roosts could facilitate the transmission of EBLV-1 between geographically separated breeding populations [19]. This statement is also supported by a study focused on RABV in the Americas, where *Myotis* species have been found to harbor a diverse range of RABV variants, suggesting that increased contact between species increases viral transmission [73]. Breeding colonies are a good site to understand the intraspecies transmission of antibodies via intra-uterine transfer, or viral transmissions as a result of biting or daily communal grooming via antibodies intra-uterine transfer [116] or biting during daily grooming [19,117]. Nerveless, characterizing EBLV-1 dynamics in juvenile bats is difficult due to unequal equilibration between them and adults and the chance that mothers can transfer antibodies to them via the placenta or during lactation [118]. The most efficient intra- and interspecific lyssavirus transmission route is during the mating period, due to aggressive male behaviour when defending territory against other males and during mating with females [19]. In support of this assertion, most cases of bat rabies in Europe peak in August and September [19,107,119].

Bats naturally infected with RABV have the virus in their nasal mucosa, leading to the proposition that airborne transmission of RABV between bats living in enclosed areas is possible [120,121,122]. Laboratory experiments showed that aerosols of the RABV virus were successfully overcome by bats but were fatal for the majority of experimental mice [123]. Johnson et al. [124] conducted a similar experiment with EBLV-2 where mice were intranasally inoculated and two of them developed the disease between 16 and 19 days post-infection. In addition, four cases of human rabies infection via aerosol were reported between 1956 and 1977 in a USA cave [124]. 

### 5.2. Other Vertebrates Cross-Species Transmission

Due to the adequately taken measures for terrestrial mammal rabies eradication in Europe [125], bats are probably the most important potential lyssavirus reservoir for humans in Europe [87]. Even so, transmission is rare; the most recent case of bat-human spillover was in southwest central France in 2019 when a human male died from EBLV-1 (https://www.zmescience.com/ecology/animals-ecology/extremely-rare-case-of-death-from-bat-rabies-in-france/), one human died from EBLV-2 in Finland in 1985, and a third in the UK in 2002 [69]. Due to growing urbanization, pets present an expected intermediate host for bat-to-human lyssavirus transmission, and both EBLV-1 and WCBV have been found in cats in France [42] and Italy [53,126]. Experimental data suggests that the infectious dose for lyssaviruses is low and certainly some human infections, especially those involving transmission from bats, support this finding [127]. 

### 5.3. Within the Contact Zone of the Palearctic and Ethiopian Realms—Potential Scenarios

Heretofore, Phylogroup II was only known from the African continent in two bat families: Pteropodidae and Hipposideridae [28,128,129]. LBV was isolated from the Straw-coloured fruit bat (*Eidolon helvum*) and Egyptian fruit bat (*Rousettus aegyptiacus*), and SHIBV from the Striped leaf-nosed bat (*Macronycteris vittatus*).

**Figure 3:**
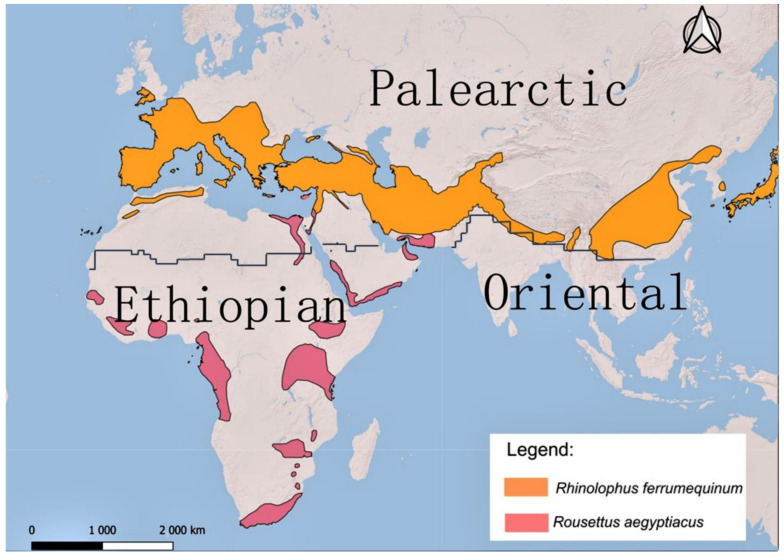
Zoogeographical distributions of *Rousettus aegyptiacus* and *Rhinolophus ferrumequinum* according to IUCN and their contact zone between the Ethiopian and Palearctic realms.

Compared with other Pteropodids, *Rousettus aegyptiacus* has the northernmost distribution, reaching Cyprus and southern Turkey [130], and roosts in a variety of underground sites [131] with other Palearctic species [132]. As a rule, cave-dwelling bats form large summer and winter colonies [93] where cross-species pathogen transmission is common [133]. A recent study [134] has shown that the families Rhinolophidae and Pteropodidae were a common factor in cross-species transmission of β-coronaviruses between continents due to their close phylogenetic relationship. This might also be applicable across the contact zones of different zoogeographic realms, e.g., Africa (Ethiopian realm) and Europe (Palearctic realm). Considering that the distributions of *Rousettus aegyptiacus* and *Rhinolophus ferrumequinum* overlap in the Southeastern zone of the Palearctic on the border with the Ethiopian realm (Figure 3), the successful transfer of lyssaviruses from Phylogroup II via the Egyptian fruit bat to other cave-dwelling species, e. g., *R. ferrumequinum*, could be a plausible scenario due to their close phylogenetic relationship within the suborder Yinpterochiroptera [16]. Furthermore, phylogeny can act as a biotic factor driving the occurrence of RNA virus cross-species transmission between closely related host species [135]. For example, *R. aegyptiacus* and various species in the genus *Rhinolophus* are known to be successful virus reservoirs of variety of β-coronavirus, *Marburgvirus*, *Henipavirus*, *Orthorubulavirus*, *Pararubulavirus*, and *Lyssavirus* [14,28,119].

Additional molecular investigations are needed to solve the relationship between Phylogroup II and the other phylogroups. This is an important consideration since Phylogroup II is spread within the families Pteropodidae and Hipposideridae, both of which are within the suborder Yinpterochiroptera and closely related to the family Rhinolophidae, which includes cave-dwelling species widely distributed across Europe. Taking into account the close phylogenetic relationship between these bat families, the spread of the lyssavirus Phylogroup II to the Palearctic realm is a possible scenario within the contact zone with the Ethiopian realm. 

## 6. Conclusions

Bat lyssaviruses in Europe belong to two phylogroups: I and highly divergent lyssaviruses. The phylogeny of the lyssaviruses is closely related to the phylogeography of Palearctic bat species. EBLV-1 has been detected in species in the superfamilies Yangochiroptera and Yinpterochiroptera, which demonstrates the virus plasticity between highly divergent bat lineages. In contrast, EBLV-2, KBLV, and BBLV have limited distributions in Europe, with their reservoirs restricted to Myotis spp. Phylogenetic relationships within Phylogroup I demonstrate that EBLV-1 and DUVV are closely related and that spillover between different bat families at a large distance from each other is possible (Europe—Africa), while the second closest group, EBLV-2, KBLV, and BBLV, has a restricted distribution in just one bat genus. The existing rabies vaccines developed for RABV eradication are effective against lyssaviruses from Phylogroup I but offer little to no protection against phylogroup II and the most divergent unclassified lyssaviruses.

Living under the shadow of the recent COVID-19 pandemic, the detection and prevention of future pathogens is of crucial importance. The newly discovered bat lyssaviruses (BBLV, LLEBV, KBLV, DBV, TWBLV-2, and MBLV) and vesiculoviruses from the USA (SDRV1 and SDRV2) and China (YSBV, TYBV, and QZBV) warn us that the Rhabdoviridae represent an unexplored pathogen pool with new and yet to be described viruses potentially adverse to human health. 

## Data Availability

Not applicable.

## Supplementary Materials

The following supporting information can be downloaded at: https://www.mdpi.com/article/10.3390/pathogens12091089/s1, Table S1. Complete N+P+M+G+L coding regions reference sequences from GenBank used in the analysis. Virus names are: Rabies virus (RABV), Aravan virus (ARAV), Australian bat lyssavirus (ABLV), Bokeloh bat lyssavirus (BBLV), Duvenhage virus (DUVV), European bat lyssavirus 1 (EBLV-1), European bat lyssavirus 2 (EBLV-2), Gannoruwa bat lyssavirus (GBLV), Irkut virus (IRKV), Khujand virus (KHUV), Lagos bat virus (LBV), Mokola virus (MOKV), Shimoni bat virus (SHIBV), Kotalahti bat lyssavirus (KBVL), Divača bat lyssavirus (DBLV), and Lyssavirus Formosa, which includes Taiwan bat lyssavirus 1 (TWBLV-1) and Taiwan bat lyssavirus 2 (TWBLV-2).
