# Supplementary material for: Phylogeographic Aspects of Bat Lyssaviruses in Europe: A Review
Source: Pathogens. 2023 Aug 27;12(9):1089. doi: 10.3390/pathogens12091089 (PMC10534866; doi:10.3390/pathogens12091089)
Supplement: Supplementary file 1 [file pathogens-12-01089-s001.zip › pathogens-2538731-supplementary.pdf]

Table S1. Complete N+P+M+G+L coding regions reference sequences from GenBank used in the analysis. Virus names are: Rabies virus (RABV), Aravan virus (ARAV), Australian bat lyssavirus (ABLV), Bokeloh bat lyssavirus (BBLV), Duvenhage virus (DUVV), European bat lyssavirus 1 (EBLV-1), European bat lyssavirus 2 (EBLV-2), Gannoruwa bat lyssavirus (GBLV), Irkut virus (IRKV), Khujand virus (KHUV), Lagos bat virus (LBV), Mokola virus (MOKV), Shimoni bat virus (SHIBV), Kotalahti bat lyssavirus (KBVL), Divača bat lyssavirus (DBLV), and Lyssavirus Formosa, which includes Taiwan bat lyssavirus 1 (TWBLV-1) and Taiwan bat lyssavirus 2 (TWBLV-2).

Virus species	Geographical origin	Year	Host	GenBank reference	Reference
RABV	USA	1986	Baby Hamster Kidney (BHK-21)	NC_001542	Tordo et al. [88]
ARAV	Kyrgyzstan	2003	*Myotis blythi*	NC_020808	Kuzmin et al. [80]
ABLV	Australia	1998	*Saccolaimus flaviventris*	NC_003243	Gould et al. [89]
BBLV	Germany	2010	*Myotis nattererii*	NC_025251	Freuling et al. [50]
DUVV	South Africa	1971	*Homo sapiens*	NC_020810	Delmas et al. [82]
EBLV-1	Germany	1968	*Eptesicus serotinus*	NC_009527	Marston et al. [90]
EBLV-2	United Kingdom	2002	*Homo sapiens*	NC_009528	Marston et al. [90]
GBLV	Sri Lanka	2015	*Pteropus giganteus*	NC_031988	Gunawardena [86]
IRKV	Russia	2003	*Murina leucogaster*	NC_020809	Kuzmin et al. [72]
KHUV	Tajikistan	2003	*Murina leucogaster*	NC_025385	Kuzmin et al. [79]
LBV	Senegal	1985	*Eidolon helvum*	NC_020807	Delmas et al. [82]
MOKV	sub-Saharan Africa	1996	*Felis catus*	NC_006429	Le Mercier et al. [91]
SHIBV	Kenya	2009	*Hipposideros commersoni*	NC_025365	Kuzmin et al. [87]
KBVL	Finland	2017	*Myotis brandti*	LR994545	Calvelage et al. [32]
DBLV	Slovenia	2023	*Myotis capaccinii*	OQ428158	Černe et al. [35]
TWBLV-1	Taiwan	2018	*Pipistrellus abramus*	ON437590	Hu et al. [33]
TWBLV-2	Taiwan	2018	*Nyctalus plancyi velutinus*	ON437589	Hu et al. [33]
